# Supplementary material for: Structural validation and assessment of AlphaFold2 predictions for centrosomal and centriolar proteins and their complexes
Source: Commun Biol. 2022 Apr 5;5:312. doi: 10.1038/s42003-022-03269-0 (PMC8983713; doi:10.1038/s42003-022-03269-0)
Supplement: Supplementary file 2 — Supplementary Material [file 42003_2022_3269_MOESM2_ESM.pdf]

Supplementary Information

Structural validation and assessment of AlphaFold2 predictions for centrosomal and centriolar proteins and their complexes.

van Breugel, M., Rosa e Silva, I., Andreeva, A.

Supplementary Figure 1

a)

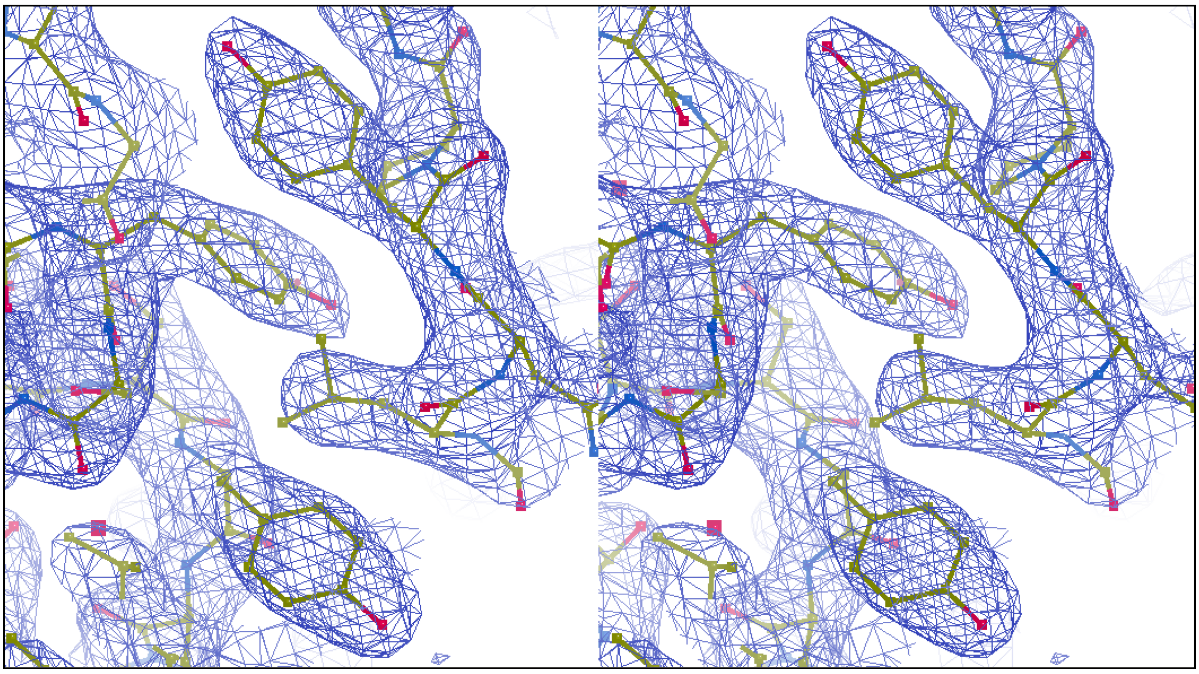

b)

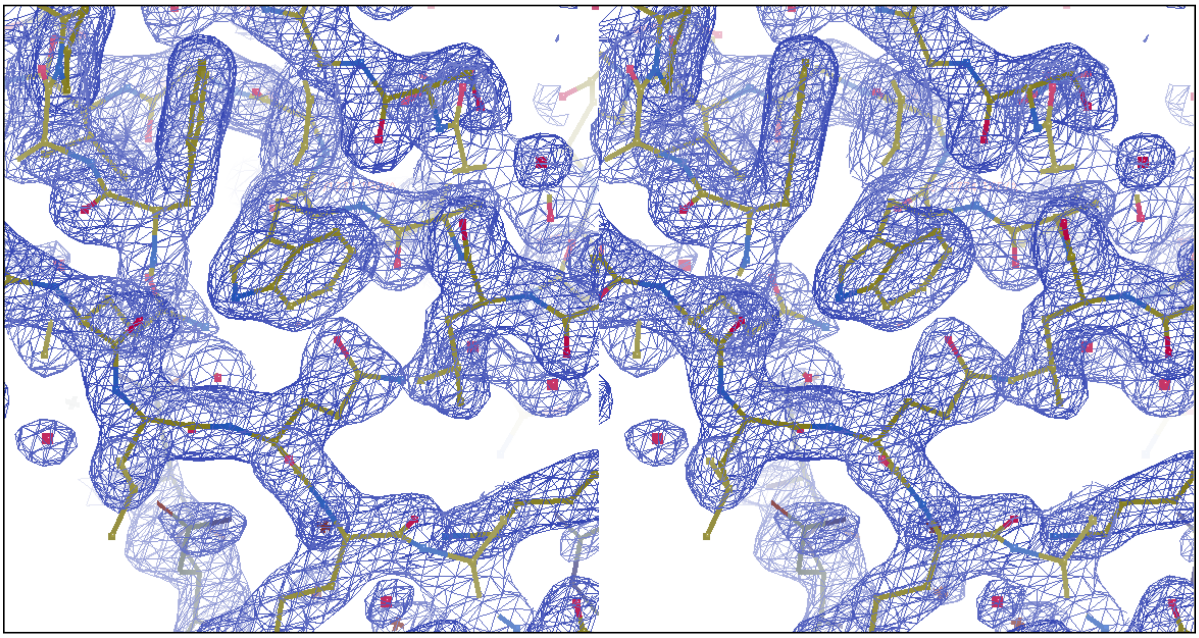

Supplementary Figure 1. Stereo view of the electron density maps of the CEP44 CH and the CEP192 Spd2 domain structure.
a) Side-by-side stereo view (wall-eyed) of a section of the 2Fo-Fc electron-density map of the CEP44 CH domain structure in an iso-mesh representation (blue) at a contour level of σ=1.5. The structure model is overlayed in a stick presentation (yellow). **b)** As in panel a, but for the CEP192 Spd2 domain structure.

Supplementary Figure 2

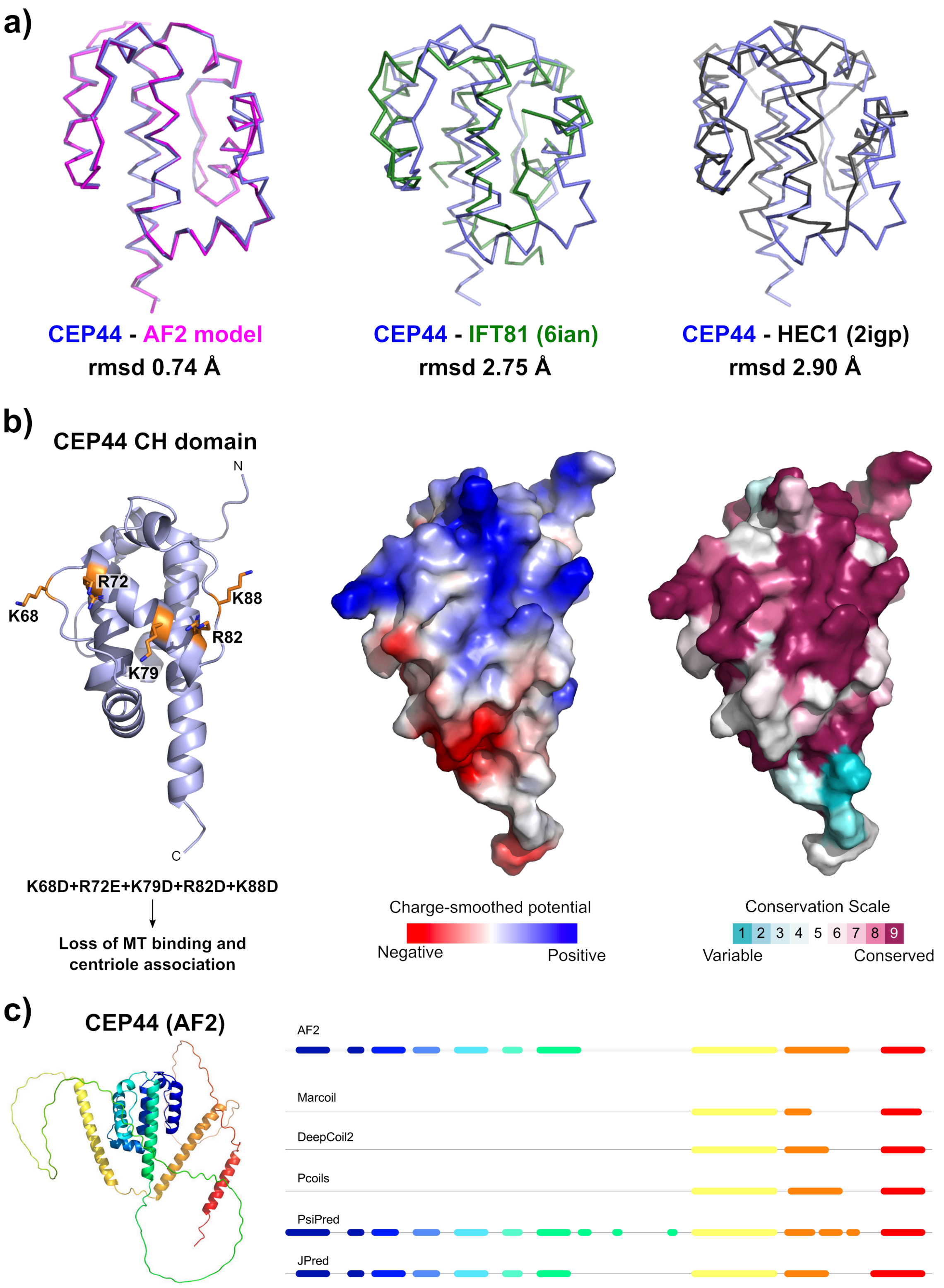

Supplementary Figure 2. The AF2-predicted CEP44 CH domain structure is remarkably similar to the experimentally determined structure.

a) Backbone superposition of the experimentally determined CEP44 CH domain structure with the AF2-predicted CEP44 CH domain structure or the most similar structures in the PDB as determined by the top hits in a HHpred search [(1)]. **b)** The CEP44 CH domain contains surface exposed, highly conserved basic residues that are crucial for microtubule binding and centriole association of CEP44. Ribbon or surface representation of the structure of CEP44's CH domain as predicted by AF2. In the ribbon representation, the basic residues that were found to abolish microtubule binding and centriole association of CEP44 [(2)] are shown as sticks (in orange) and are labeled. Surface representations are coloured by the charge-smoothed vacuum potential from negative (red) to positive (blue) or colour-coded according to the ConSurf conservation scores from variable (cyan) to conserved (burgundy). **c)** AF2 model of full length CEP44 (AF-Q9C0F1-F1-model_v1), rainbow-coloured from blue (N-terminus) to red (C-terminus), together with a comparison of its secondary structure elements with those predicted by the indicated secondary structure and coiled coil prediction programs. Rounded, coloured bars designate alpha helices.

Supplementary Figure 3

a)

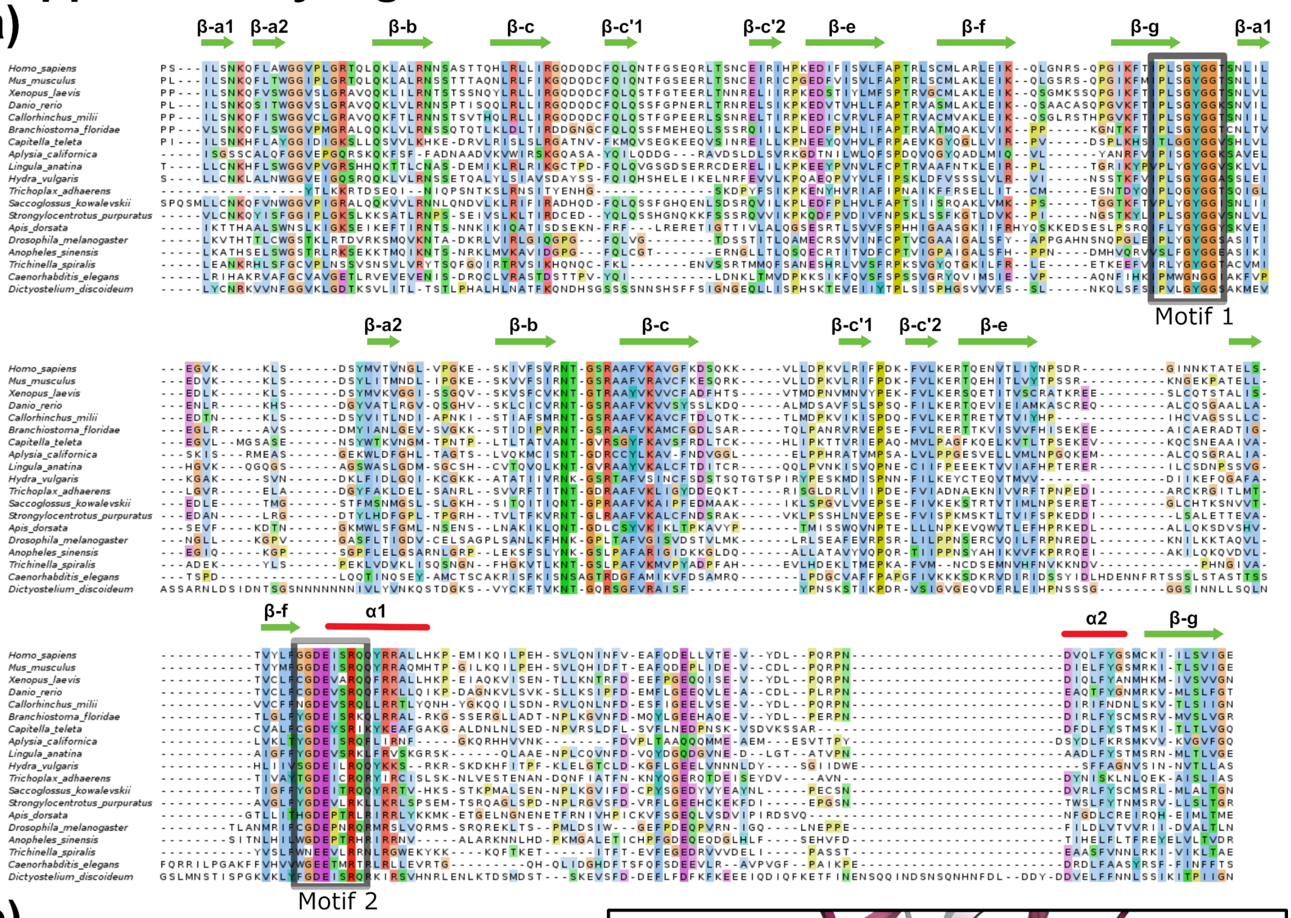

b)

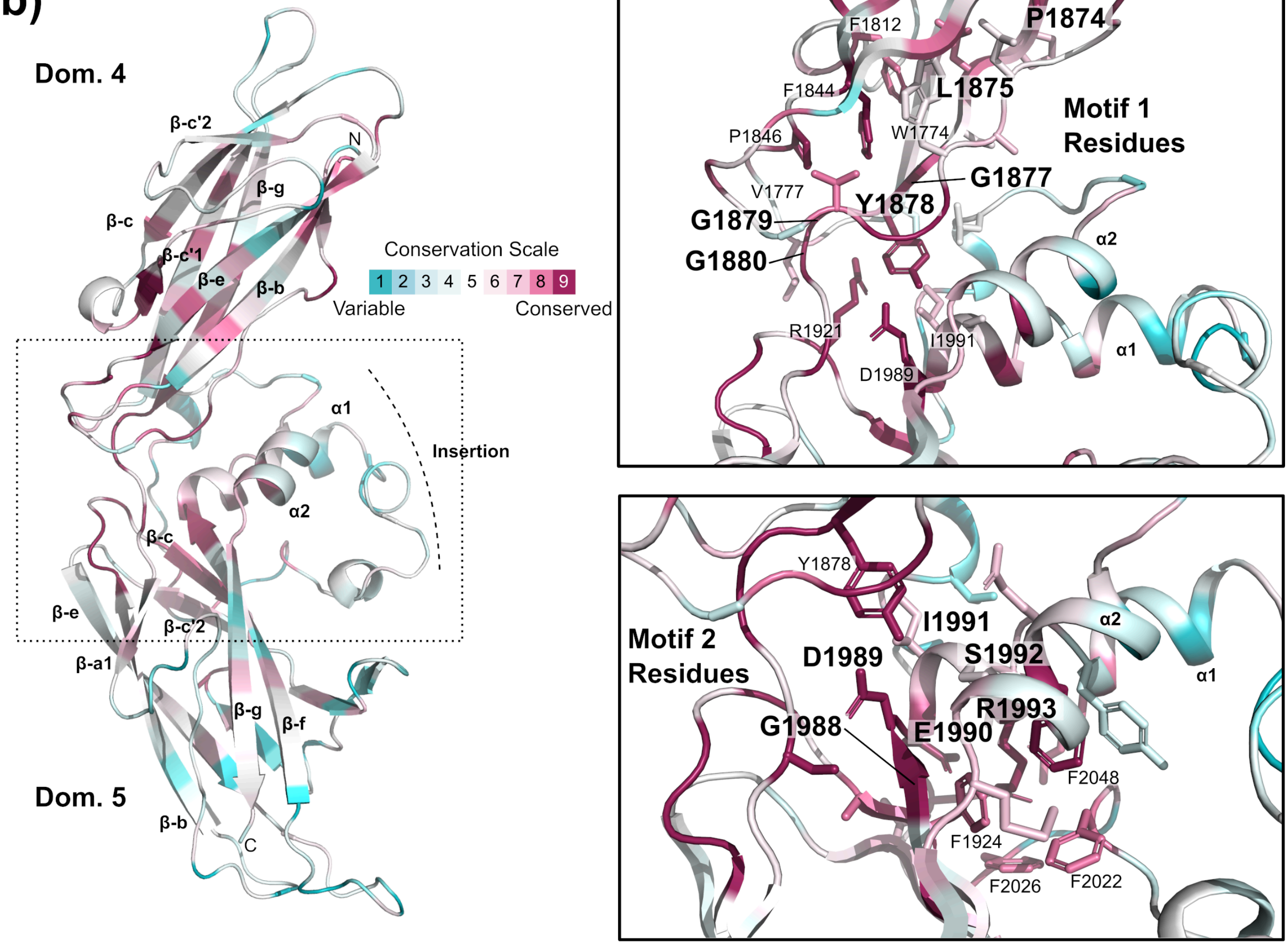

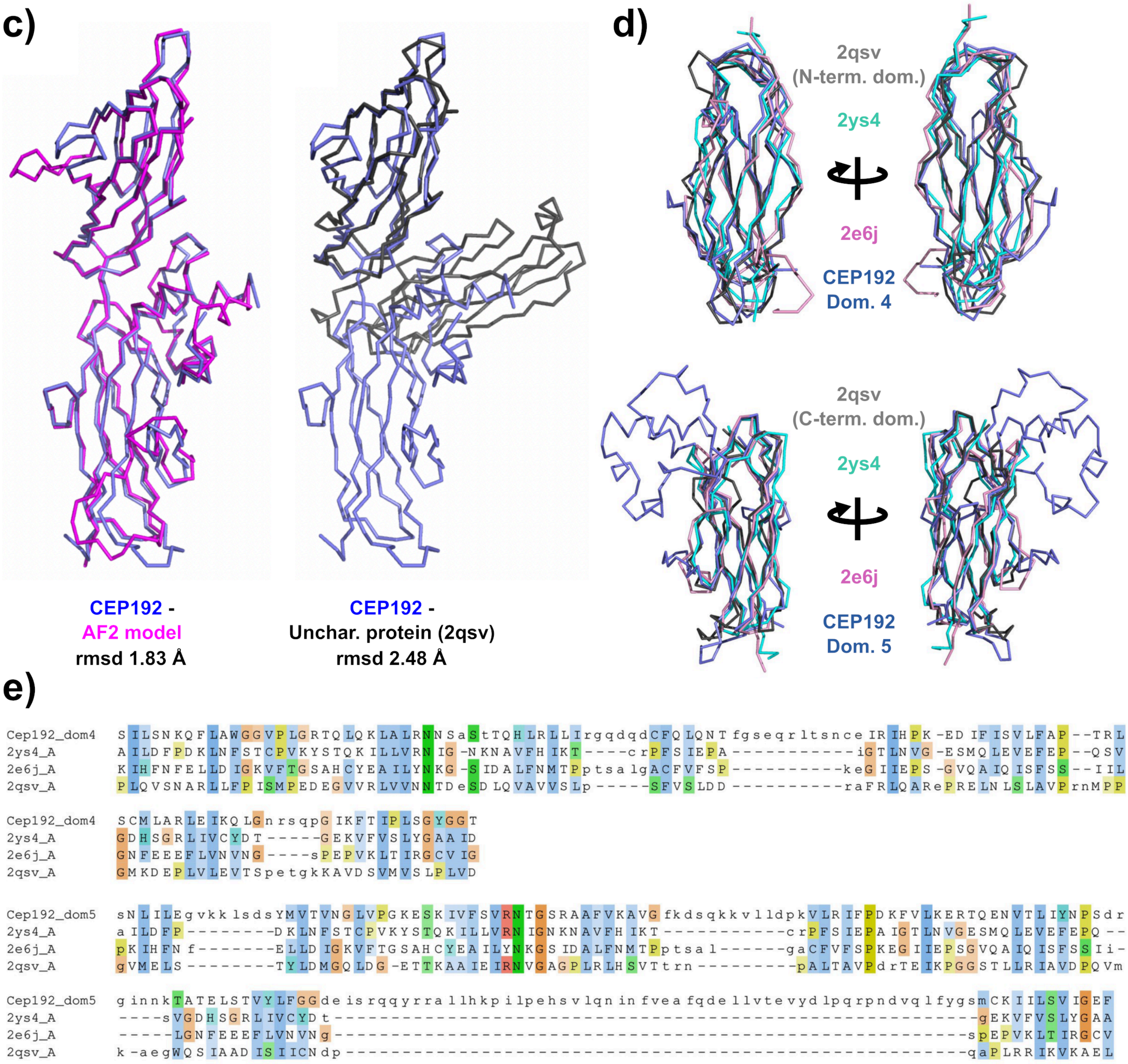

Supplementary Figure 3. Analyses of the experimentally determined CEP192 Spd2 domain structure in comparison to the AF2 structure prediction and to the most similar structures in the PDB.

a) Multiple sequence alignment of CEP192 / Spd2 homologues spanning their Spd2 domain. The secondary structure elements of the two subdomains of the Spd2 domain in human CEP192 (PapD-like domain 4 and 5) are indicated above the alignment. Its beta strands are labeled according to the strand nomenclature of Bullock and colleagues [3]. The two most conserved motifs (Motif 1 and 2) found in the Spd2 domain are boxed. **b)** Ribbon representation of the experimentally determined high-resolution structure of the Spd2 domain of human CEP192 consisting of PapD-like domain 4 and 5. The structure is colour-coded according to the ConSurf conservation scores from variable (cyan) to conserved (burgundy). The dotted box indicates the region shown magnified with selected sidechains displayed as sticks and labelled. Residues labelled in bold are part of Motif 1 or Motif 2 as indicated. **c)** Backbone superposition of the experimentally determined CEP192 Spd2 domain structure with the AF2-predicted CEP192 Spd2 domain structure or with its most similar structure in the PDB as determined by a HHpred search [1]. **d)** Backbone superposition of the two subdomains of the experimentally determined CEP192 Spd2 domain structure with their most similar structures in the PDB as determined by HHpred search [1]. **e)** The corresponding sequence alignments of the superpositions shown in panel d.

Supplementary Figure 4

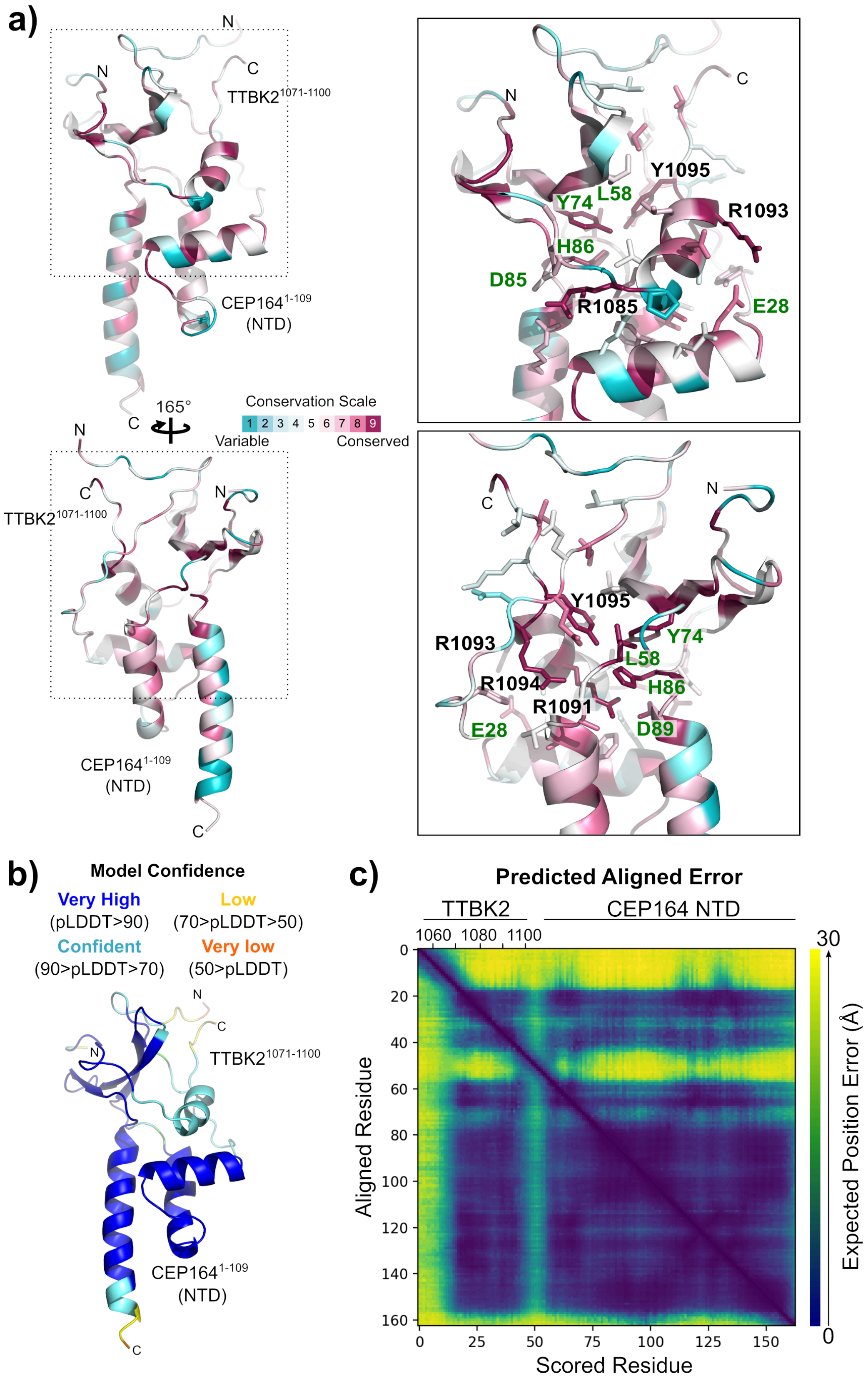

Supplementary Figure 4. Analyses of the AF2-predicted CEP164$^{1\text{-}109}$-TTBK2$^{1071\text{-}1100}$ complex structure. **a)** The predicted additional CEP164$^{1\text{-}109}$-TTBK2$^{1071\text{-}1100}$ interface is highly conserved. Ribbon representation of the AF2-predicted CEP164$^{1\text{-}109}$-TTBK2$^{1071\text{-}1100}$ complex structure, colour-coded according to the ConSurf conservation scores from variable (cyan) to conserved (burgundy). Rotation, as indicated. The regions in dashed boxes are shown as magnified views on the right with selected residues depicted as sticks and labeled. Black labels indicate TTBK2, green labels CEP164 residues. **b-c)** The CEP164$^{1\text{-}109}$-TTBK2$^{1071\text{-}1100}$ complex structure is predicted with high confidence. **b)** Ribbon representation of the AF2-predicted CEP164$^{1\text{-}109}$-TTBK2$^{1071\text{-}1100}$ complex structure, colour-coded by the pLDDT values. **c)** The Predicted Aligned Error of the AF2-predicted CEP164-TTBK2 complex structure showing the high prediction confidence of the CEP164-TTBK2 contact region. Above the plot, the corresponding TTBK2 amino acid residue numbers and the CEP164$^{1\text{-}109}$ (NTD) position are indicated.

Supplementary Figure 5

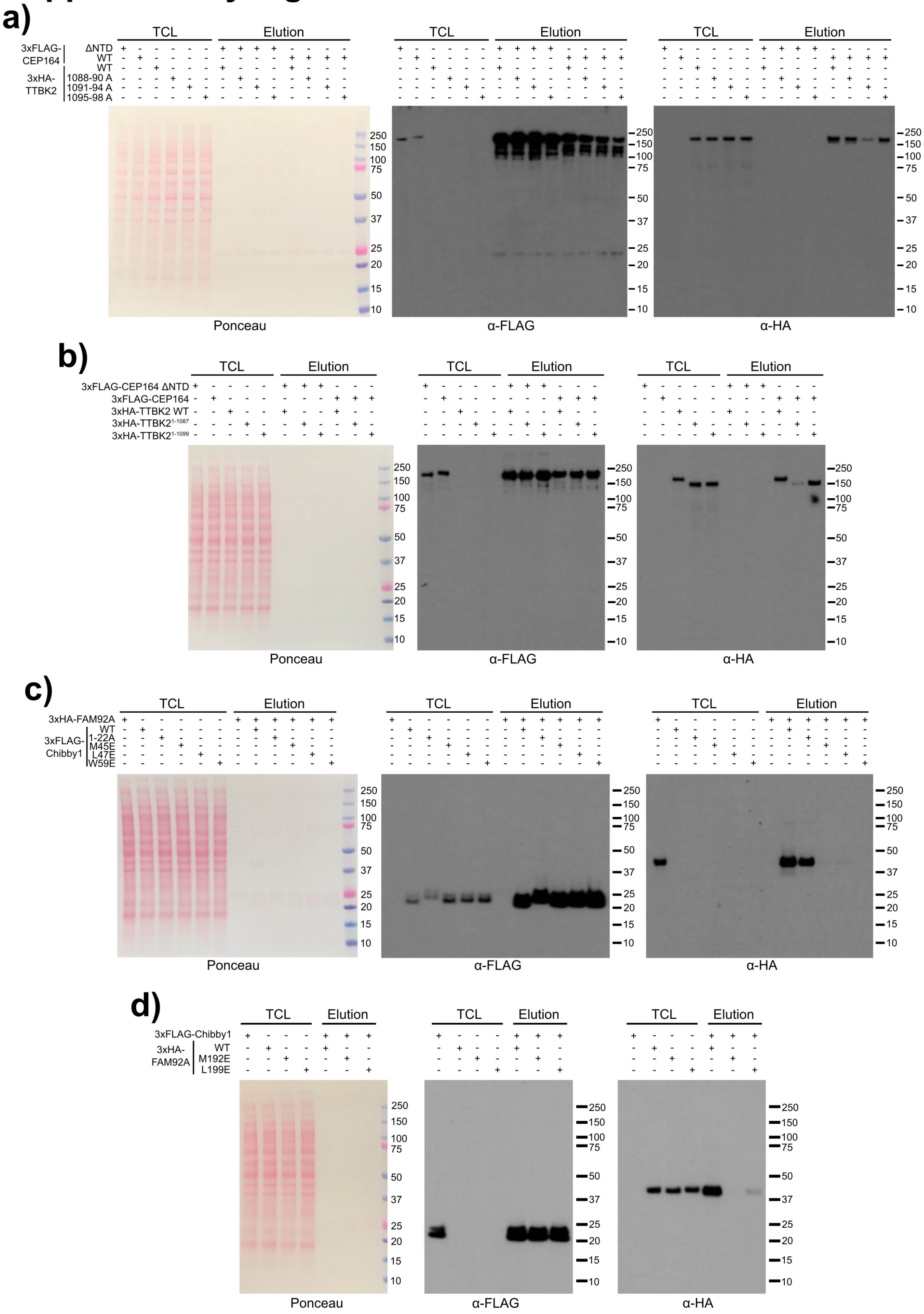

Supplementary Figure 5. Uncropped western blots of the CEP164-TTBK2 and Chibby1-FAM92A pull-down experiments.

a) The uncropped western blot of the CEP164-TTBK2 pulldown experiment shown in Figure 2c. **b)** The AF2-predicted additional interface between TTBK2$^{1088\text{-}1099}$ and the CEP164 N-terminal domain (NTD) is important for efficient complex formation. Western blot showing a pull-down experiment with lysates from cells expressing the indicated 3xFLAG-tagged CEP164 or 3xHA-tagged TTBK2 constructs. The TTBK2 proline rich region that engages the WW domain of CEP164-NTD ends at residue 1084. TCL: Total cell lysate. **c)** The uncropped western blot of the Chibby1-FAM92A pulldown experiment shown in Figure 3b. **d)** The uncropped western blot of the Chibby1-FAM92A pulldown experiment shown in Figure 3c.

Supplementary Figure 6

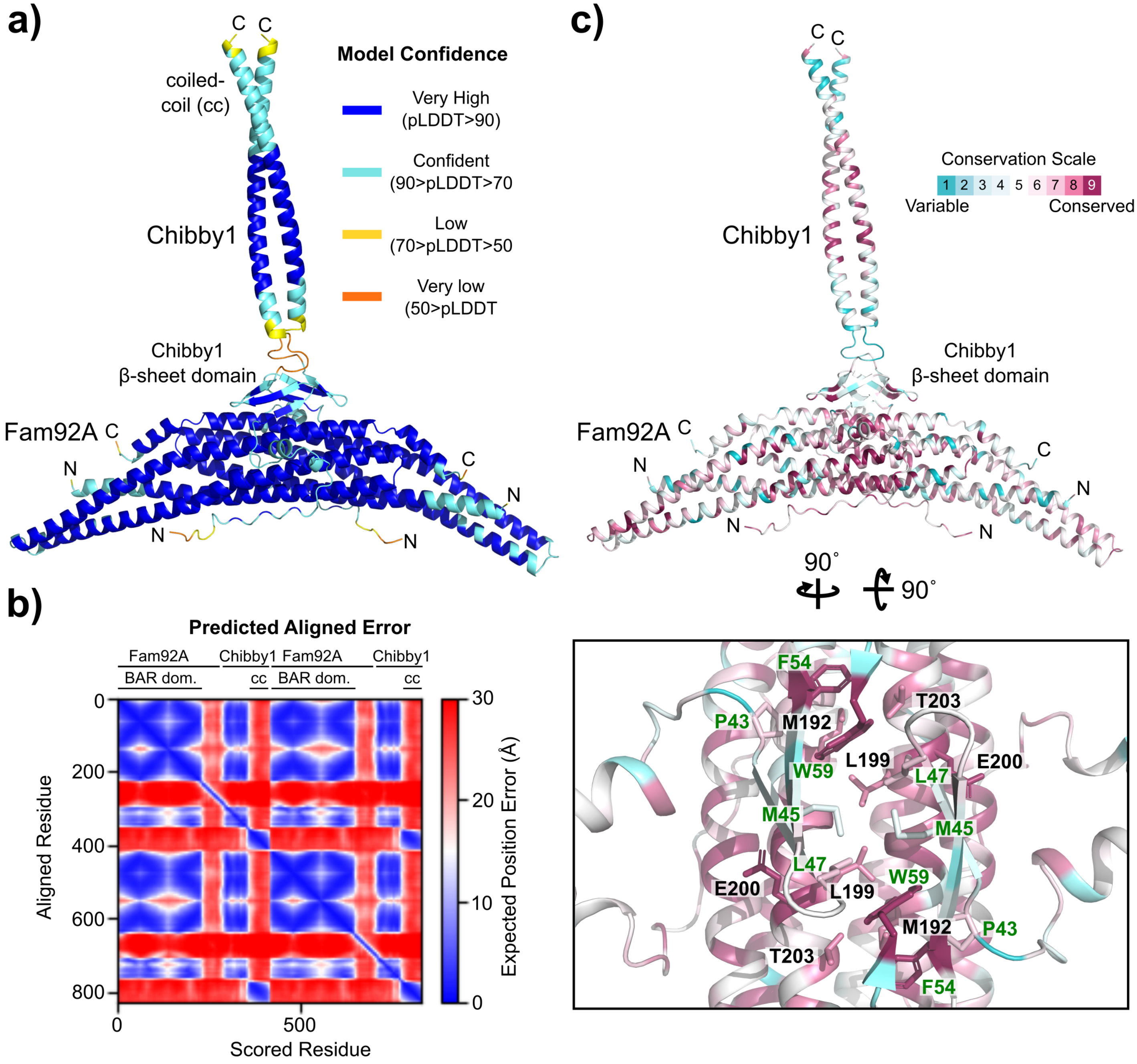

Supplementary Figure 6. The interface of the Chibby1-FAM92A complex is conserved and predicted with high confidence by AF2.

a-b) The Chibby1-FAM92A complex structure is predicted with high confidence. **a)** Ribbon representation of the AF2-predicted Chibby1-FAM92A complex structure, colour-coded by the pLDDT values. **b)** The Predicted Aligned Error of the AF2-predicted Chibby1-FAM92A complex structure showing the high prediction confidence of the Chibby1-FAM92A contact region. Above the plot, the corresponding FAM92 and Chibby1 positions in the fusion protein are indicated. cc designates the coiled-coil domain of Chibby1. **c)** The predicted Chibby1-FAM92A interface is conserved. Ribbon representation of the AF2-predicted Chibby1-FAM92A complex structure, colour-coded according to the ConSurf conservation scores from variable (cyan) to conserved (burgundy). Shown boxed is a detailed view on the interface between the beta-sheet domain of Chibby1 and the BAR domain of FAM92A. Selected sidechains are shown as sticks and are labeled. The rotation angles to obtain this view are indicated. Black labels indicate FAM92A, green labels Chibby1 residues.

Supplementary References

1. Söding J, Biegert A, Lupas AN. (2005). The HHpred interactive server for protein homology detection and structure prediction. Nucleic Acids Res. 33:W244-8.

2. Atorino ES, Hata S, Funaya C, Neuner A, Schiebel E. (2020). CEP44 ensures the formation of bona fide centriole wall, a requirement for the centriole-to-centrosome conversion. Nat Commun. 11(1):903.

3. Bullock, T.L., Roberts, T.M., and Stewart, M. (1996). 2.5 A resolution crystal structure of the motile major sperm protein (MSP) of Ascaris suum. Journal of molecular biology *263*, 284-296.
