# Supplementary material for: Structural validation and assessment of AlphaFold2 predictions for centrosomal and centriolar proteins and their complexes
Source: Commun Biol. 2022 Apr 5;5:312. doi: 10.1038/s42003-022-03269-0 (PMC8983713; doi:10.1038/s42003-022-03269-0)
Supplement: Supplementary file 3 — Reporting Summary [file 42003_2022_3269_MOESM3_ESM.pdf]

nature portfolio

Corresponding author(s): Mark van Breugel

Last updated by author(s): Feb 8, 2022

Reporting Summary

Nature Portfolio wishes to improve the reproducibility of the work that we publish. This form provides structure for consistency and transparency in reporting. For further information on Nature Portfolio policies, see our Editorial Policies and the Editorial Policy Checklist.

Statistics

For all statistical analyses, confirm that the following items are present in the figure legend, table legend, main text, or Methods section.

n/a	Confirmed	
☐	☒	The exact sample size (*n*) for each experimental group/condition, given as a discrete number and unit of measurement
☒	☐	A statement on whether measurements were taken from distinct samples or whether the same sample was measured repeatedly
☒	☐	The statistical test(s) used AND whether they are one- or two-sided<br>*Only common tests should be described solely by name; describe more complex techniques in the Methods section.*
☒	☐	A description of all covariates tested
☒	☐	A description of any assumptions or corrections, such as tests of normality and adjustment for multiple comparisons
☒	☐	A full description of the statistical parameters including central tendency (e.g. means) or other basic estimates (e.g. regression coefficient) AND variation (e.g. standard deviation) or associated estimates of uncertainty (e.g. confidence intervals)
☒	☐	For null hypothesis testing, the test statistic (e.g. *F*, *t*, *r*) with confidence intervals, effect sizes, degrees of freedom and *P* value noted<br>*Give P values as exact values whenever suitable.*
☒	☐	For Bayesian analysis, information on the choice of priors and Markov chain Monte Carlo settings
☒	☐	For hierarchical and complex designs, identification of the appropriate level for tests and full reporting of outcomes
☒	☐	Estimates of effect sizes (e.g. Cohen's *d*, Pearson's *r*), indicating how they were calculated

Our web collection on statistics for biologists contains articles on many of the points above.

Software and code

Policy information about availability of computer code

Data collection	X-ray diffraction datasets were collected at the Diamond Light Source (UK) at beamlines I24 and I03 using the data acquisition software installed at these beamlines (data collection at 9.5.2018 and 23.6.2018, respectively)
Data analysis	X-ray crystallography data was processed using iMOSFLM (version 7.2.1 and 7.2.2), AIMLESS (version 0.6.2 and 0.7.1), CRANK2 (version 2.0.281) and SHELX C/D/E (version 2016/1 / 2013/2 / 2018/1) and refined with PHENIX (version 1.17.1_3660) and REFMAC (version 5.8.0267). The software used for structure comparisons and analyses is listed in the Methods section.

For manuscripts utilizing custom algorithms or software that are central to the research but not yet described in published literature, software must be made available to editors and reviewers. We strongly encourage code deposition in a community repository (e.g. GitHub). See the Nature Portfolio guidelines for submitting code & software for further information.

Data

Policy information about availability of data

All manuscripts must include a data availability statement. This statement should provide the following information, where applicable:

- Accession codes, unique identifiers, or web links for publicly available datasets
- A description of any restrictions on data availability
- For clinical datasets or third party data, please ensure that the statement adheres to our policy

The data that support the findings of this study are presented in this manuscript and are available from the corresponding author upon request. The coordinates and structure factors of the CEP44 and the CEP192 structure have been deposited at the PDB under code 7PT5 and 7PTB, respectively.

Field-specific reporting

Please select the one below that is the best fit for your research. If you are not sure, read the appropriate sections before making your selection.

☒ Life sciences ☐ Behavioural & social sciences ☐ Ecological, evolutionary & environmental sciences

For a reference copy of the document with all sections, see nature.com/documents/nr-reporting-summary-flat.pdf

Life sciences study design

All studies must disclose on these points even when the disclosure is negative.

Sample size	Pulldown experiments showed a drastic reduction in binding by the mutants compared to the control. This was replicated in an independent experiment and a sample size of two for these experiments was therefore deemed sufficient.
Data exclusions	No data was excluded
Replication	Pulldown experiments were performed in independent duplicates (n=2) and replication was successful and consistent.
Randomization	Biochemical pulldown experiments were performed with tube orders randomly scrambled during the processing steps.
Blinding	Due to the nature of structural studies no blinding was done with X-ray crystallography experiments. The pull-down experiments did not require blinding as the results showed dramatic differences in binding by the mutants compared to the wild-type that do not require subjective interpretation.

Reporting for specific materials, systems and methods

We require information from authors about some types of materials, experimental systems and methods used in many studies. Here, indicate whether each material, system or method listed is relevant to your study. If you are not sure if a list item applies to your research, read the appropriate section before selecting a response.

Materials & experimental systems

n/a	Involved in the study	
☐	☒	Antibodies
☐	☒	Eukaryotic cell lines
☒	☐	Palaeontology and archaeology
☒	☐	Animals and other organisms
☒	☐	Human research participants
☒	☐	Clinical data
☒	☐	Dual use research of concern

Methods

n/a	Involved in the study	
☒	☐	ChIP-seq
☒	☐	Flow cytometry
☒	☐	MRI-based neuroimaging

Antibodies

Antibodies used	Western Blotting: anti-FLAG (Sigma, F1804), anti-HA (gift from Dr Hegde (MRC-LMB, Cambridge, UK))
Validation	Cell lysates showed only bands in Western blots when cells were transfected with the correspondingly tagged constructs (3xFLAG or 3xHA). These bands showed the expected molecular weights with all the different constructs used.

Eukaryotic cell lines

Policy information about cell lines

Cell line source(s)	The Flip-In T-REx-293 cell line was a gift from Dr Hegde (MRC-LMB, Cambridge, UK)
Authentication	The Flip-In T-REx-293 cell line was not authenticated by me. Authentication was deemed unnecessary as the cell line was only used for pull-down experiments.
Mycoplasma contamination	Cell line tested negative for Mycoplasma contamination
Commonly misidentified lines (See ICLAC register)	*Name any commonly misidentified cell lines used in the study and provide a rationale for their use.*
